# Studies on the growth performance of different broiler strains at high altitude and evaluation of probiotic effect on their survivability

**DOI:** 10.1038/srep46074

**Published:** 2017-04-11

**Authors:** Sahil Kalia, Vijay K. Bharti, Deepak Gogoi, Arup Giri, Bhuvnesh Kumar

**Affiliations:** 1 Defence Institute of High Altitude Research (DIHAR), DRDO, C/o- 56 APO, Leh-Ladakh, (J&K), 194101, India

## Abstract

Identification of appropriate breeds of broilers and development of new feed additives is required for the development of poultry industry at high altitude. Therefore, this experiment was conducted first to identify the suitable broiler strain for this region. One week old chicks (150) from three broiler strains, i.e. Vencobb, RIR cross-bred, and Hubbard were randomly selected and divided equally into three groups. All the chicks were provided the same basal diet. The body weight gain and feed: gain responses were significantly (P < 0.05) improved in RIR cross-bred. Mortality was also observed lower in RIR cross-bred. Thereafter, the second trial was conducted in RIR cross-bred to evaluate the effect of probiotic supplementation (T1@ 9 gm/kg feed, T2@ 18 gm/kg feed) on their performance and mortality. No significant differences (P > 0.05) were observed in weight gain, feed intake, feed: gain, and water intake among the three groups, however, mortality from ascites and coccidiosis was reduced in probiotic treated groups. Hence, our results suggest that RIR cross-bred is suitable for rearing in high altitude regions and probiotic supplementation has no beneficial effects on production performance of broilers at high altitude. However, probiotic supplementation indicated lesser loss due to mortality of birds.

The last two decades the broiler industry in India has made a good deal of progress. However, if we compare the growth performance of broiler poultry birds reared at cold, arid high altitude Himalayas with the performance of poultry birds in the rest of the country the picture is very gloomy. The major constraint for this difference in the growth could be attributed to stressful environmental conditions in this region, which are characterized by extreme climatic conditions, hypobaric hypoxia, unavailability of suitable broiler breeds and feed^1^. Due to all these reasons, poultry entrepreneurship produces a low return of income to local farmers. Since the extreme climatic condition is not suitable for commonly available broiler strains that are developed for plain areas. Therefore, there is a need to carry out experimental trials to find suitable broiler strains for this region, which are adapted to this climate and fast growing. Adaptive broiler strains could be less susceptible to high altitude mediated adverse problems and therefore will have better growth rate.

In the poultry industry, feed constitutes the major input that decides the profit margin. However, there is no feed industry, which is producing commercial poultry feed for high altitude chicken. So, available commercial broiler feed has no nutritional composition to meet the maintenance and productive requirement of birds at high altitude. Therefore, unless suitable feed formulation is developed, their genetic potential will not be expressed fully even of most suitable broiler strain. Different kinds of feed additives viz. antibiotics, prebiotic, probiotic, etc. are frequently supplemented in broiler feed for their better growth and survivability^2^,^3^. Antibiotic growth promoters (AGP) have been very useful in enhancing the growth performance and improving the feed efficiency in livestock^4^. However, the excessive use of AGP in animal diet enhanced the development of antibiotic resistant bacterial strains, which via transfer through the food chain imposes negative effects in animal and human health^5^. As a result, interest in the effects of probiotics on animal health and their performance has been increased.

Probiotics or direct-fed microbial (DFM) are the living microorganisms which when administrated in the host animal, they improve the intestinal microbial balance and give beneficial effects^6^,^7^,^8^,^9^. Probiotic protects animal health against pathogens, enhanced immune response, reduced antibiotic use, and show a high index of safety^10^,^11^,^12^. Hence, it was hypothesized that probiotic may improve the production and health of chicken at high altitude condition through the reduction in pathogenic gut microbial load and increase in nutrient utilization^13^,^14^. Efficacy study must be conducted to evaluate the beneficial effects of probiotics in target species/animal categories under different rearing conditions^15^. However, no study has been undertaken to investigate the effect of probiotic on growth performance and survivability of broiler chicken at high altitude. *Bacillus coagulans* and *Saccharomyces cerevisiae* have been used as a source of probiotics for improving growth performance and feed conversion ratio of broiler chickens due to their property of competitive exclusion which reduces the microbial load of harmful microbe^16^,^17^,^18^. Hence, commercially available probiotic (Biobloom^T^, M/S Zydus Animal Health Limited, India) which contains above microbes was fed to the experimental birds. This probiotic has been extensively used in commercial livestock rearing^19^,^20^,^21^.

Therefore, in the present study, we first evaluated the growth performance of different strains of broiler chicken and further, we investigated the effect of commercially available probiotic supplementation on the performance of most adaptable broiler strain at high altitude.

## Methods

### Location of the experiment

The experiment was approved by the Institutional Animal Ethics Committee of DIHAR and all the methods were performed according to the guidelines of animal experimentation. The place of the experiment is situated at 3500 meters above mean sea level in Ladakh, India. The present experiment was carried out under a deep litter system in the solar poultry house having a stacking density of 0.80 square feet per bird in 4 × 2 feet of pen size (10 birds/pen). The ambient temperature of the house was maintained at 25–32 °C by using local Bukhari (a heating device). This housing system overcomes extreme temperature effect prevalent at Ladakh.

### Experimental trial I

**Figure 1:**
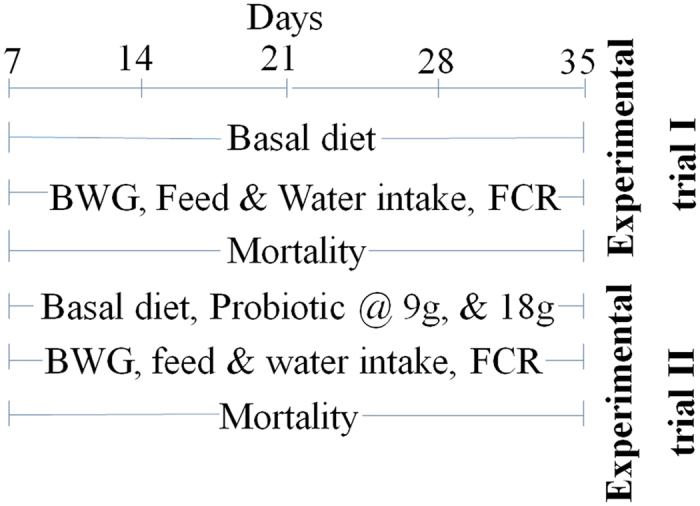
A scheme of the protocol for experimental trials. Birds fed with basal diet in experimental trial I from days 7 to 35. For experimental trial II, birds fed with basal diet (Control) or diet containing probiotic @ 9 g/kg (T-I) or @18 g/kg (T-II) from days 7 to 35.

The study was conducted in two experimental trials. The experimental protocol is described in Fig. 1. In the first trial, the growth performance of different broiler strains was compared to find out suitable broiler strain for high altitude. For this study, a total of 50 no. one week old chicks from each breed of broiler strains, i.e. Vencobb, RIR cross-bred, and Hubbard brought from a commercial hatchery of plain areas were randomly selected. Thereafter, they were divided into 3 groups of 5 replicates with 10 chicks in each replicate according to completely randomized design so that mean values differ as little as possible. All the birds were given the same basal diet, included a starter diet (21.56% protein, 12.97 MJ/Kg ME, 1.02% calcium, and 0.48% phosphorus) from 1^st^ day to 21^st^ day and a finisher diet (19.31% protein, 13.38 MJ/Kg ME, 0.94% calcium, and 0.40% phosphorus) from 22^nd^ day to the 35^th^ day. Basal diet used in this study was a standard in-house commercial formulation developed by DIHAR, which is used since last 10 years. Individual feed ingredients and other micronutrients are procured and ration formulated as per the standard feed formula to meet the maintenance and production requirements of birds under high altitude condition^22^,^23^,^24^. The ingredients and chemical composition of basal diet are presented in Table 1. The experiment was conducted from 7^th^ to 35^th^ days of broiler age. The chicks were fed *ad libitum*. On 7^th^day, all the chicks were vaccinated for Newcastle disease. All the chickens were weighed individually at every week interval up to 35^th^ days of age. Throughout the experiment, water and feed intake were measured. Feed: gain (FCR) was calculated by dividing feed intake by body weight gain of chick.

### Experimental trial II

Thereafter, a second trial was conducted, where best-performing broiler strain (among the above-mentioned three strains) was supplemented with probiotic (Biobloom^T^, M/S Zydus Animal Health Limited, India) mixed basal diet. Due to low availability of day old chicks at high altitude it was difficult to perform a study on a large number of chicks. Therefore, under the second trial limited number (150 no.) of one week old commercial broiler chicks brought from a commercial hatchery of plain areas were randomly assigned into 3 groups in 5 replicates. So, 10 chicks in each replicate and having 50 birds in each group according to completely randomized design, however this much number of birds did not affect our findings. Birds in the control group were fed the basal diet, whereas the two treatment groups fed the basal diet supplemented with probiotic T1 @ 9 gm/kg feed (0.45 × 10^9^ CFU of *Bacillus coagulans* and 13.5 × 10^9^ CFU of *Saccharomyces cerevisiae*) and T2 @ 18 gm/kg feed (0.9 × 10^9^ CFU of *Bacillus coagulans* and 27 × 10^9^ CFU of *Saccharomyces cerevisiae)*. Each gram of probiotic contains 50 × 10^6^ CFU of *Bacillus coagulans* and 1500 × 10^6^ CFU of *Saccharomyces cerevisiae*. The study was conducted from 7^th^ to 35^th^ days of broiler age. Vaccination, feeding program and composition of the basal diet was similar to experimental trial I. Birds died during both trials were examined for ascites and coccidiosis on post-mortem examination.

### Statistical analysis

Statistical analysis was performed by using SPSS 17.0 statistical software package as per the standard procedure of one-way ANOVA. The data were expressed as a mean ± standard error. The means of different experimental groups were compared with Duncan’s multiple range test. Significant statistical difference was calculated at P < 0.05.

## Results

### Growth performance of each strain of broiler chickens in experimental trial I

In this study, we evaluated the growth performance of three different broiler strains at high altitude using body weight gain (BWG), feed intake, feed: gain (FCR), and water intake parameters. The mean values of BWG, feed intake, feed: gain (FCR), and water intake are presented in Table 2. We observed that, at the age of 35 days, RIR cross-bred fed with basal diet reached an average BWG of 288.22 ± 3.55 g. In contrast, Vencobb and Hubbard had an average BWG of 254.39 ± 3.81 g and 250.47 ± 3.63 g, respectively (Table 2). Feed intake was significantly higher (P < 0.05) in Hubbard strain (1091.33 ± 6.13) followed by RIR cross-bred (1037.34 ± 6.43) and Vencobb (1003.45 ± 6.90). Moreover, birds of RIR cross-bred, Vencobb, and Hubbard strains had an average feed: gain (FCR) of 3.60 ± 0.05, 3.94 ± 0.03, and 4.36 ± 0.04, respectively (Table 2). Similarly, birds of RIR cross-bred, Vencobb, and Hubbard strains had an average water intake of 1780.03 ± 6.35, 1785.06 ± 6.53, and 1810.84 ± 6.11, respectively. Collectively, BWG and FCR data illustrated that RIR cross-bred had improved growth performance in comparison to Vencobb and Hubbard strains at high altitude.

### Mortality in each strain of broiler chickens in experimental trial I

Next, we recorded the number of mortalities in each broiler strain in experimental trial I. As shown in Table 3, the mortality rate was recorded highest (30%) in Hubbard strain followed by Vencobb (22%) and RIR cross-bred (16%). Post-mortem examination of birds revealed 14%, 10%, and 6% mortality induced from ascites and 10%, 8%, and 4% mortality induced from coccidiosis in Hubbard, Vencobb, and RIR cross-bred, respectively.

### Growth performance of RIR cross-bred chickens in experimental trial II

Next, we examined the effect of dietary supplementation of probiotic on growth performance of RIR cross-bred birds (as it performed better in the experimental trial I) in experimental trial II using BWG, feed intake, feed: gain (FCR), and water intake parameters. The mean values of BWG, feed intake, feed: gain and water intake are presented in Table 4. Probiotic supplementation at two different concentrations did not show any beneficial effect on their growth performance as compared to control group. Moreover, feed and water intake did not differ between the groups. Consistently, we did not observe any difference in the feed: gain value in between three experimental groups (Table 4). Collectively, BWG and FCR data illustrated that supplementation of probiotic in broiler diet had no improvement on their growth performance at high altitude.

### Economics and mortality in RIR cross-bred chickens in experimental trial II

In parallel, we recorded the number of mortalities in RIR cross-bred birds and economics was calculated in experimental trial II. As shown in Table 5, the mortality rate was recorded highest (30%, total 15 birds out of 50) in the control group followed by T1 (20%, total 10 birds out of 50) and T2 (16%, total 8 birds out of 50) groups. Post-mortem examination of birds revealed 10%, 8%, and 6% mortality induced from ascites and 16%, 6%, and 4% mortality induced from coccidiosis in control, T1, and T2 groups, respectively.

At the end of the experiment, economics were calculated based on rearing cost of 50 no. of birds. Since other expenditure remained constant only additional probiotic supplementation cost was included with the feed cost. Probiotic supplementation increased the return due to the reduction in mortality as compared to control group (Table 5).

## Discussion

Identification of the appropriate strains of broiler birds, which can cope with stress full environmental conditions of high altitude, are required to promote poultry farming in this part of world^25^,^26^. In this study, we examined the growth performance and survivability of fast growing Vencobb and Hubbard broiler strains along with a RIR cross-bred strain which has abilities for adapting in stressful environment conditions^26^,^27^. Our finding of this study indicated very low growth rate in all the broiler strains that might be due to the adverse effect of high altitude stress. These findings were compared with the growth rate and FCR of these chicken strains reared at lowland, which indicated the significant reduction in growth rate and poor FCR at high altitude^24^,^28^,^29^.

High altitude coupled with hypoxia affects the body metabolism, which causes negative energy balance due to the reduction in energy intake and an increase in energy expenditure, so the overall growth rate is decreased^30^. This disturbance in energy balance leads to decrease in body mass by intestinal malabsorption and with an increase in the catabolism of muscle protein and body fats. Among the three strains, RIR cross-bred was performed better than Vencobb and Hubbard strains. This fall in the growth performance of Vencobb and Hubbard strain birds might be due to their high metabolic rate as these birds are fast growing which requires more oxygen at the tissue level to sustain fast growth^31^. However, due to hypobaric hypoxia, there is an imbalance between oxygen intake and oxygen requirement which leads to ascites and reduced growth rate^25^,^32^.

Fall in the immunity in birds under hypoxic conditions makes them susceptible to numerous microbial diseases, including coccidiosis^7^,^8^,^30^. In this study, we noticed high mortality in Hubbard and Vencobb strain with symptoms of ascites and coccidiosis, which indicates less adaptability of these two strains for this region. In contrary to these two strains, RIR cross-bred exhibited less mortality due to ascites and coccidiosis. This might be due to their hardiness nature and less metabolic rate as compared to fast-growing Vencobb and Hubbard^30^. Hence, dual-purpose RIR cross-bred of chickens may be helpful at high altitude for rearing.

In the present study, we also evaluated the effect of dietary supplementation of probiotic on growth performance and survivability of RIR cross-bred (a best-performing broiler strain among the three strains) birds under experimental trial II. Interestingly, we did not observe any beneficial effect of probiotic on growth performance of RIR cross-bred. On the contrary to our results, previous coworkers^16^,^17^ reported significant improvement in broiler growth and feed conversion ratio after supplementation of *Bacillus coagulans* and *Saccharomyces cerevisiae* as probiotic in broiler diet. The contradictory findings of the present study may be attributed to either insufficient number of *Bacillus coagulans* and *Saccharomyces cerevisiae* strain used in this study or unsuitable kinds of these microbes present in the probiotic^33^. However, the present dose was higher than the dose of plain areas i.e. 0.6 × 10^8^ CFU/gram for *Bacillus coagulans* per Kg feed^16^ and 2 × 10^6^ CFU/gram for *Saccharomyces cerevisiae*^17^, respectively. So, it is concluded that different kinds of microbes may be suitable for poultry birds at high altitude. Although probiotic containing specific microbes may not help in complete amelioration of adverse effect of high altitude stress, but may show some improvement in growth and nutrient utilization.

The reduction in mortality rate from coccidiosis in probiotic treated birds might be due to the presence of surface adhesions (mucus-binding proteins) on the surface of probiotic bacteria^34^ which mediate the attachment of probiotics with intestinal epithelial cells (IECs) and this interaction between probiotic bacteria and IECs may inhibit the colonization of pathogenic bacteria in intestinal mucosa^35^ through competitive exclusion mechanism. Competitive exclusion by probiotic bacteria is based on its higher affinity to compete for available nutrients and adhesion sites on intestinal mucosa as compared to gastrointestinal pathogen^36^. As coccidiosis in chickens is caused by *Eimeria* parasite which needs to invade inside the host cell for its replication and therefore, it must have to adhere IECs. However, *Bacillus-*based probiotics in this study may compete with *Eimeria* oocysts for adhesion sites on epithelial cells and might retard its penetration through the chicken intestinal mucosal barrier. This, consequently, inhibits its replication and improves resistance in chickens against Eimeria parasites^37^. In addition, probiotic bacteria might induce the secretion of mucins^38^ and β-defensins^39^ from epithelial cells, which improve intestinal mucosal barrier functions and it also secretes antimicrobial substances such as bacteriocins, which have a strong inhibitory effect against gastrointestinal pathogens^40^. Moreover, probiotic bacteria have the ability to modulate innate as well as the adaptive intestinal mucosal immunity of poultry birds^41^,^42^.

The used probiotics, i.e. *Saccharomyces cerevisiae* and *Bacillus coagulans* have immunomodulatory properties in broilers and it has already been reported by previous coworkers Bai *et al*.^17^ and Panda *et al*.^43^. Increased phagocytosis by macrophages and improved oxidative burst activity of avian heterophils has been reported after administration of *Bacillus*-based probiotics^44^,^45^,^46^ in broilers diet. *Bacillus-*based probiotics enhance the activity of natural killer cells^47^ and induce the production of secretory IgA in Payer’s patches which help in maintaining of mucosal humoral immune response^48^. Probiotic bacteria have also been shown to alter intestinal mucosal pro and anti-inflammatory cytokine expressions^49^. Intestinal inflammation is mainly accompanied with an imbalance in intestinal microflora, leading to the generation of pro-inflammatory mediators^50^. Probiotics can suppress intestinal inflammation through down-regulation of toll-like receptor (TLR) signaling pathway expression, which leads to decrease in the production of local pro-inflammatory tumor necrosis factor α (TNF α) and inhibition of NF-kB signaling in enterocytes^51^. In addition, probiotics also modulate the intestinal immune response by inducing dendritic cells (DCs) maturation and mature DCs induce T cell differentiation to regulatory T cells (T_reg_) that ultimately increase the number of anti-inflammatory cytokine IL-10^52^.

Moreover, probiotics suppress T cell proliferation which leads to decrease in the production of pro-inflammatory cytokines from T cells^53^. Therefore, it is possible that the modification of broilers gastrointestinal microflora with *Bacillus*-based probiotics in this study might stimulate mucosal innate and adaptive immune response against *Eimeria* parasites and other gastrointestinal pathogens. Our finding is in agreement with the report of Dalloul *et al*.^37^ where the supplementation of *Bacillus*-based probiotic increased the intestinal mucosal immunity against *Eimeria* parasite in broilers. Furthermore, reduction in the mortality due to ascites in broilers might be due to the beneficial effects of probiotic on body metabolism and due to the hardiness nature of RIR cross-bred birds^2^. This finding is in accordance with the report of Saffar and Khajali (2010)^54^ where the probiotic supplementation prevents mortality in broilers caused by ascites.

Moreover, the reduction in mortality increased the net return of income, in spite of no effect on body weight gain in probiotic treated groups. The efficacy of probiotic depends upon dose, types of microbes present in the probiotics, kinds of the gut microbial population, types of basal diet ingredients used in the diet formulation, prevalent stress condition, etc.^15^,^55^. Since at high altitude, stresses are much higher and kinds of the gut microbial population are also unknown. Hence, further investigation on the utility of different microbial strains at high altitude would be helpful to identify suitable probiotic strains and their dose optimization. This will help with comprehensive knowledge on the probiotic usefulness on improving the growth, nutrient utilization, and survivability at high altitude as comparable to lowland or plain areas.

In conclusion, unsatisfactory growth performance of broiler birds in this study at high altitude can be overcome by identifying appropriate strains of broiler chicken having more adaptability under hypoxic conditions. RIR cross-bred has shown better growth and survivability, may be due to less metabolic rate, and hence would be suitable for high altitude. Net returns revealed a higher profit in the probiotic-supplemented group. Therefore, dietary supplementation of probiotic as a feed additive in broiler diet could be a valuable source for improvement in their survivability rate and save the loss due to high mortality at high altitude. However, more additional knowledge is required on probiotics containing suitable microbial population, dose, and kinds of gut microflora in the gastrointestinal tract before their selection.

## Additional Information

**How to cite this article**: Kalia, S. *et al*. Studies on the growth performance of different broiler strains at high altitude and evaluation of probiotic effect on their survivability. *Sci. Rep.* **7**, 46074; doi: 10.1038/srep46074 (2017).

**Publisher's note:** Springer Nature remains neutral with regard to jurisdictional claims in published maps and institutional affiliations.

## Figures and Tables

**Table 1:** Ingredients and chemical composition of basal diet.

Ingredients (% diet)	Starter diet(7–21 day)	Finisher diet(22–35 day)
Maize	59.00	58.00
Soyabean (Solvent extracted)	33.18	21.12
Soyabean (Full fat)	—	9.58
Soyabean oil	2.00	2.55
Fish Meal	2.15	—
Wheat bran	—	5.08
Salt (Nacl)	0.15	0.15
Limestone	1.50	1.50
Dicalcium phosphate	1.50	1.50
Lysine	0.13	0.13
Methionine	0.19	0.19
Vitamin & Mineral premix*	0.20	0.20
Total	100	100
Calculated composition		
Protein (%)	21.56	19.31
ME (MJ/Kg)	12.97	13.38
Calcium (%)	1.02	0.94
Phosphorus (%)	0.48	0.42

^*^Nutrition value per Kg of vitamin and mineral premix contains 140000 IU of vitamin A, 70 mg of vitamin E, 3000 IU of vitamin D3, 4 mg of vitamin K, 3 mg of thiamine, 10 mg of vitaminB2, 8 mg of vitamin B6, 0.04 mg of vitamin B12, 48 mg of niacin, 20 mg of calcium d-pantothenate, 500 mg of choline chloride, 0.20 mg of biotin, 1.8 mg of folic acid, 80 mg of manganese, 70 mg of zinc, 50 mg of iron, 10 mg of copper, 3 mg of iodine, 0.4 mg of selenium, and 0.2 mg of cobalt.

**Table 2:** Growth performance of each strain of broiler chickens in the experimental trial I.

Attributes	Vencobb	RIR cross-bred	Hubbard
Body weight at 7^th^ day (gm/bird)	40.31 ± 0.44	41.09 ± 0.46	40.03 ± 0.36
BWG (gm/bird)			
7–21 days of age	90.19^a^ ± 2.00	106.13^b^ ± 2.38	93.65^a^ ± 2.30
22–35 days of age	164.20^a^ ± 3.75	182.09^b^ ± 3.39	156.82^a^ ± 3.25
7–35 days of age	254.39^a^ ± 3.81	288.22^b^ ± 3.55	250.47^a^ ± 3.63
Feed intake (gm/bird)			
7–21 days of age	422.40^a^ ± 4.75	429.52^a^ ± 4.19	449.71^b^ ± 4.23
22–35 days of age	581.05^a^ ± 6.68	607.82^b^ ± 6.58	641.62^c^ ± 6.45
7–35 days of age	1003.45^a^ ± 6.90	1037.34^b^ ± 6.43	1091.33^c^ ± 6.13
Feed: gain (gm:gm)			
7–21 days of age	4.68^b^ ± 0.03	4.05^a^ ± 0.02	4.80^b^ ± 0.03
22–35 days of age	3.54^b^ ± 0.04	3.34^a^ ± 0.04	4.09^c^ ± 0.06
7–35 days of age	3.94^b^ ± 0.03	3.60^a^ ± 0.05	4.36^c^ ± 0.04
Water intake (ml/bird)			
7–21 days of age	834.58 ± 5.71	825.83 ± 5.37	840.25 ± 5.59
22–35 days of age	950.48 ± 6.46	954.20 ± 6.43	970.59 ± 6.61
7–35 days of age	1785.06 ± 6.53	1780.03 ± 6.35	1810.84 ± 6.11

Means bearing the different superscripts ^(a, b, c)^ in a row differ significantly (P < 0.05). BWG = body weight gain. FCR = (feed intake)/(BWG).

**Table 3:** Mortality in each strain of broiler chickens in the experimental trial I.

	Vencobb	RIR cross-bred	Hubbard
Total mortality (%)	22.00 (11/50)	16.00 (8/50)	30.00 (15/50)
Mortality due to ascites (%)	10.00 (5/50)	06.00 (3/50)	14.00 (7/50)
Mortality due to coccidiosis (%)	08.00 (4/50)	04.00 (2/50)	10.00 (5/50)
Mortality due to other reasons (%)	04.00 (2/50)	06.00 (3/50)	06.00 (3/50)

All the birds died during experimental trial I, has undergone post-mortem examination. Mortality due to ascites and coccidiosis were observed during the post-mortem examination.

**Table 4:** Effect of dietary probiotic on growth performance of RIR cross-bred chickens in experimental trial II.

	Control	T1	T2
Body weight at 7^th^ day (gm/bird)	39.06 ± 0.39	38.86 ± 0.50	40.53 ± 0.38
BWG (gm/bird)			
7–21 days of age	109.50 ± 2.16	108.03 ± 2.41	106.72 ± 2.29
22–35 days of age	60.17 ± 1.69	57.91 ± 2.03	60.85 ± 1.83
7–35 days of age	169.67 ± 2.35	165.94 ± 2.61	167.57 ± 2.39
Feed intake (gm/bird)			
7–21 days of age	453.67 ± 5.14	451.44 ± 4.89	466.07 ± 5.07
22–35 days of age	605.11 ± 6.27	601.44 ± 6.31	596.16 ± 6.19
7–35 days of age	1058.78 ± 6.55	1052.88 ± 6.17	1062.23 ± 6.31
Feed: gain (gm:gm)			
7–21 days of age	4.14 ± 0.04	4.18 ± 0.03	4.37 ± 0.04
22–35 days of age	10.05 ± 0.07	10.39 ± 0.06	9.80 ± 0.08
7–35 days of age	6.24 ± 0.03	6.34 ± 0.03	6.34 ± 0.04
Water intake (ml/bird)			
7–21 days of age	794.58 ± 4.97	791.51 ± 5.16	787.78 ± 5.11
22–35 days of age	845.32 ± 5.59	864.58 ± 5.81	866.61 ± 5.63
7–35 days of age	1639.90 ± 5.81	1656.09 ± 6.03	1654.39 ± 5.85

Means bearing no superscript in a row did not differ significantly (P > 0.05). Control = Basal diet, T1 = Basal diet + 9 gm/kg Probiotic, T2 = Basal diet + 18 gm/kg Probiotic. BWG = body weight gain. FCR = (feed intake)/(BWG).

**Table 5:** Economics and mortality of RIR cross-bred chickens supplemented with probiotics at high altitude.

Description	Control	T1	T2
Total mortality (%)	30	20	16
Mortality due to ascites (%)	10	8	6
Mortality due to coccidiosis (%)	16	6	4
Mortality due to other reasons (%)	4	6	6
Cost of probiotic/bird (Rs.)	Nil	2	4
Cost of feed/bird (@ Rs. 25/kg) (Rs.)	26.47	26.32	26.56
Total feed cost/bird (Rs.)	26.47	28.32	30.56
Sale of per bird at 35 days (Rs.) (@ Rs. 200/kg live weight *	41.75	40.96	41.62
Loss due to mortality (Rs.)**	626.25	409.60	332.96
Total benefits per group (Rs.) #	—	216.65	293.29

Control = Basal diet, T1 = Basal diet + 9 gm/kg Probiotic, T2 = Basal diet + 18 gm/kg Probiotic. All the birds died in experimental trial II undergo post mortem examination. Mortality induced by ascites and coccidiosis were notice. * Rate of fresh chicken meat is very high at high altitude due to limited availability. **Loss due to mortality: sale cost of per bird × total number of mortality. # Total benefits per group: Loss due to mortality (Control) - Loss due to mortality (Treatment).
